# Increasing Cas9-mediated homology-directed repair efficiency through covalent tethering of DNA repair template

**DOI:** 10.1038/s42003-018-0054-2

**Published:** 2018-05-31

**Authors:** Eric J. Aird, Klaus N. Lovendahl, Amber St. Martin, Reuben S. Harris, Wendy R. Gordon

**Affiliations:** 1 Department of Biochemistry, Molecular Biology, and Biophysics, University of Minnesota, Minneapolis, MN 55455 USA; 2 Center for Genome Engineering, University of Minnesota, Minneapolis, MN 55108 USA; 3 Masonic Cancer Center, Institute for Molecular Virology, University of Minnesota, Minneapolis, MN 55455 USA; 4 Howard Hughes Medical Institute, University of Minnesota, Minneapolis, MN 55455 USA

## Abstract

The CRISPR-Cas9 system is a powerful genome-editing tool in which a guide RNA targets Cas9 to a site in the genome, where the Cas9 nuclease then induces a double-stranded break (DSB). The potential of CRISPR-Cas9 to deliver precise genome editing is hindered by the low efficiency of homology-directed repair (HDR), which is required to incorporate a donor DNA template encoding desired genome edits near the DSB. We present a strategy to enhance HDR efficiency by covalently tethering a single-stranded oligodeoxynucleotide (ssODN) to the Cas9-guide RNA ribonucleoprotein (RNP) complex via a fused HUH endonuclease, thus spatially and temporally co-localizing the DSB machinery and donor DNA. We demonstrate up to a 30-fold enhancement of HDR using several editing assays, including repair of a frameshift and in-frame insertions of protein tags. The improved HDR efficiency is observed in multiple cell types and target loci and is more pronounced at low RNP concentrations.

## Abstract (web summary)

Eric Aird et al. present a strategy to increase the efficiency of homology-directed repair in CRISPR/Cas9-mediated genome editing. They show that tethering a single-stranded oligodeoxynucleotide to the ribonucleoprotein complex using a fused HUH endonuclease increases editing efficiency by up to 30-fold.

## Introduction

The ability of CRISPR-Cas9 to specifically target sites in the genome to produce a double-stranded break (DSB) has made it a critical tool in genome editing^1,2^. Mammalian cells repair the DSB predominantly through two pathways: non-homologous end joining (NHEJ) or homology-directed repair (HDR)^3^. The more frequent NHEJ pathway results in the formation of small insertions or deletions (indels) at the DSB site, while the alternative HDR pathway can be utilized to insert exogenous DNA sequences into the genome^3,4^. For many applications, HDR is desired but is crippled by the low efficiency of recombination. Thus, various approaches have been developed to boost HDR frequency. Small molecules inhibiting NHEJ or upregulating HDR pathways have been reported to enhance HDR^5–7^. Another method to increase HDR efficiency is to regulate cell cycle progression or temporal control of Cas9 expression, either through small molecules or Cas9 protein fusions^8,9^. While these approaches and others have been reported to increase HDR efficiencies by as much as 10-fold, they suffer both from negative impacts on cell growth and inconsistencies between cell type and targeted loci^10^.

We reasoned that a strategy to covalently tether the DNA donor template to the Cas9-guide RNA ribonucleoprotein (RNP) could dramatically enhance HDR efficiency by guaranteeing the presence of the donor DNA at the site of the DSB with the RNP complex, thus increasing the effective concentration of the donor template at the DSB and reducing the dimensionality of the reaction. In a recently described method termed S1mplex^11^, authors used recombinant streptavidin to bridge a modified single-guide RNA (sgRNA) containing a streptavidin-binding aptamer and a biotinylated single-stranded oligodeoxynucleotide (ssODN) to deliver a linked RNP-donor DNA complex and observed enhancement in precise genome editing compared to unlinked components. However, this method, along with other recent attempts to co-deliver the donor DNA using biotin-avidin^12^ and the SNAP tag^13^, requires additional steps and expense associated with modifying the donor DNA and inclusion of additional recombinant proteins. Additionally, the S1mplex method generally suffers from decreased absolute HDR rates^11^.

We recently showed that HUH endonucleases form robust covalent bonds with specific sequences of unmodified single-stranded DNA (ssDNA) and can function in fusion tags with diverse protein partners, including Cas9^14^. Formation of a phosphotyrosine bond between ssDNA and HUH endonucleases occurs within minutes at room temperature. Tethering the donor DNA template to Cas9 utilizing an HUH endonuclease could thus be a powerful approach to create a stable covalent RNP-ssODN complex without the need for chemical modification of the ssODN, alteration of the sgRNA, or additional proteins. We demonstrate that covalent-tethering of an ssODN via HUH-Cas9 fusion proteins results in up to a 30-fold enhancement of HDR. We present relative and absolute readouts of HDR using several editing assays, including repair of a frameshift and in-frame insertions of protein tags. The improved HDR efficiency is observed in multiple cell types and target loci and is more pronounced at low RNP concentrations.

## Results

### Selective covalent attachment of ssDNA to Cas9-PCV

**Fig. 1:**
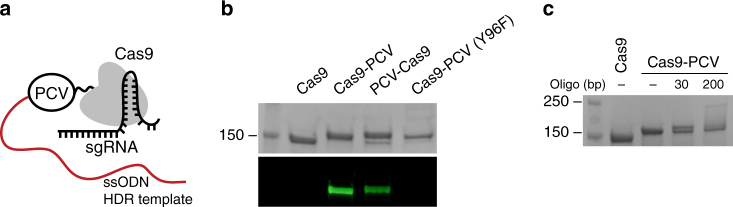
Selective covalent attachment of ssDNA to Cas9-PCV. **a** Schematic of Cas9 fused to the HUH endonuclease PCV with a covalently attached ssODN. **b** SDS-PAGE of Cas9 variants reacted with an Alexa 488 fluorescently labeled ssDNA containing the PCV recognition sequence. The top panel is the coomassie stained gel, and the bottom panel is the identical fluorescently imaged gel. PCV is fused to either the carboxyl (Cas9-PCV) or amino (PCV-Cas9) terminus of Cas9. Cas9-PCV (Y96F) represents catalytically inactive PCV (Y96F) fused to Cas9. **c** SDS-PAGE gel shift assay of Cas9 reacting with two ssDNA templates containing the PCV recognition sequence of differing lengths in a 1:1 ssDNA:Cas9 molar ratio

We first fused the Porcine Circovirus 2 (PCV) Rep protein^14,15^ to either the amino (PCV-Cas9) or carboxyl (Cas9-PCV) terminus of Cas9 (Fig. 1a). We assayed for the selective attachment of ssDNA using fluorescently labeled ssDNA containing the recognition sequence for PCV (Fig. 1b). Both PCV fused Cas9 variants are able to bind the oligonucleotide while unfused Cas9 does not. In addition, mutating the catalytic tyrosine of PCV (Y96F) predictably prevents covalent attachment of the DNA. To assess reactivity of Cas9-PCV with ssODNs used in the HDR experiments, the upwards mobility shift of the protein-DNA conjugate was monitored using SDS-PAGE (Fig. 1c). An equimolar ratio of protein to DNA resulted in >60% covalent complex formation (by gel densitometry) after 15 min at room temperature. The ability of Cas9 to cleave DNA was assessed in vitro and found to not be perturbed by the addition of PCV at either terminus (Supplementary Fig. 1).

### Covalent tethering enhances insertion of a peptide tag

**Fig. 2:**
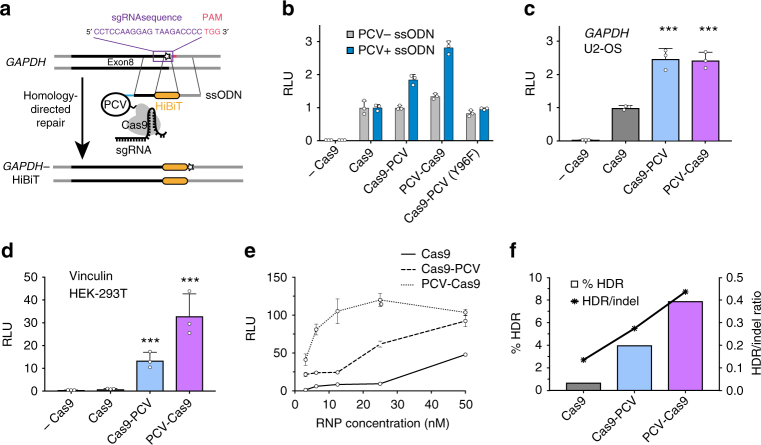
Covalent attachment of ssODN to PCV fusions of Cas9 enhances insertion of a peptide tag. **a** Schematic of split luciferase insertion. The C-terminus of nanoluciferase (HiBiT) is encoded on the 200 bp ssODN along with the 5′ PCV recognition sequence and targeted to the 3′-end of *GAPDH*. **b** Assaying luminescence using different Cas9 variants when inserting HiBiT into *GAPDH* in HEK-293T cells. PCV is fused to either the amino (PCV-Cas9) or carboxyl (Cas9-PCV) terminus of Cas9. Transfections were performed with ssODN lacking the PCV recognition sequence (PCV- ssODN) or ssODN containing the PCV recognition sequence (PCV+ ssODN). Units are displayed in relative light units (RLU) normalized to Cas9. **c** Targeting the *GAPDH* locus in U2-OS cells. **d** Targeting a locus in vinculin in HEK-293T cells using an ssODN containing the PCV recognition sequence. **e** Fold change in RLU compared to Cas9 when varying the RNP concentration with equimolar ssODN. **f** HDR frequency and HDR/indel ratio from deep sequencing of the *GAPDH* locus. Graphs represent data from one of multiple independent experiments exhibiting similar results using distinct samples. The data with error bars are shown as mean+/− SD (*n* = 3). Significance calculated using 2-tailed Student’s *t* test: **P < 0.01, ***P < 0.001

We next used a recently described luminescence activity assay (Promega; see Methods) to monitor the in-frame insertion of the 13 amino acid C-terminus of split-nanoluciferase (HiBiT) reporter into endogenous loci as a readout of HDR efficiency^16^. Adding the recombinant N-terminus (LgBiT) reconstitutes full-length nanoluciferase only in the edited cells and produces light upon addition of substrate. The intensity of light emitted is thus a relative measure of HDR efficiency. RNPs targeting the 3′-end of *GAPDH* were assembled in vitro and transfected into HEK-293T cells with or without ssODN containing the PCV recognition sequence (Fig. 2a). When using an ssODN lacking the PCV recognition sequence (PCV- ssODN), all versions of Cas9 resulted in similar luminescence levels when assayed 48 h post-transfection (Fig. 2b). However, upon addition of an ssODN containing the PCV recognition sequence (PCV+ ssODN), a substantial two to three-fold change in luminescence is observed for cells transfected with Cas9-PCV fusions. The increase in luminescence was abrogated in catalytically inactive PCV (Y96F) fused to Cas9, suggesting the specific attachment of ssODN to Cas9 via PCV is responsible for the increase in HDR frequency. This enhancement of HDR is not limited to HEK-293T cells or the particular *GAPDH* locus. We have targeted *GAPDH* in osteosarcoma U2-OS cells for HiBiT insertion and observed similar effects (Fig. 2c). We have also targeted an in-frame locus between two domains of vinculin, which is expressed at lower levels than GAPDH in HEK293T cells, and measured a significant increase in luminescence using the Cas9-PCV fusion in comparison to Cas9 alone (Fig. 2d). Furthermore, changing the strand sense of the ssODN relative to the sgRNA does not impact the HDR enhancement (Supplementary Fig. 2). Combining our methodology with the addition of SCR7, an NHEJ inhibitor, increases HDR efficiency further (Supplementary Fig. 2), suggesting covalent attachment of DNA could be combined with other published methods to create additive effects^6,7^.

Importantly, the HDR enhancement observed in the context of PCV-tagged Cas9 and co-delivery of the repair DNA cannot be recapitulated by merely increasing the Cas9 or donor DNA concentrations. RNP titrations reveal increased HDR for Cas9-PCV when compared to Cas9 regardless of the concentration (Fig. 2e). Interestingly, the enhancement is much more pronounced at lower concentrations of Cas9-PCV, where up to a 15- to 30-fold change in HDR exists. Similarly, increasing the concentration of ssODN containing the PCV recognition sequence has no effect on Cas9 HDR efficiency, while for Cas9-PCV, the maximal luminescence readout is seen when at a 1-to-1 ratio of RNP to ssODN (Supplementary Fig. 2). Taken together, the luminescence data strongly suggests the significant increase in HDR is due to covalent tethering of the DNA template to Cas9-PCV.

We used quantitative-PCR (qPCR) to confirm that the effect seen at the protein level in the luminescence assay is consistent at the DNA level. We assayed genomic DNA preparations for insertion of HiBiT. Primers were designed to amplify only in the presence of the insertion with a reference primer pair located upstream on *GAPDH* (Supplementary Fig. 3). The upstream primer for the HiBiT pair binds outside of the ssODN sequence so no amplification occurs from the ssODN itself. Using the Pfaffl method to quantitate differences in cycle threshold values^17^, we observed a consistent two-fold change in HDR efficiency between Cas9 and Cas9-PCV fusions that mirrors the luminescence results (Supplementary Fig. 3).

The efficiency of HiBiT integration was also confirmed and quantified with deep sequencing of the *GAPDH* and vinculin loci (Supplementary Fig. 4 and Supplementary Table 1). Analysis of *GAPDH* targeting showed a 5- to 11-fold change in absolute HDR efficiency with the tethered ssODN system (Fig. 2f). For instance, correct recombination of HiBiT in *GAPDH* occurred using 25 nM Cas9 at a rate of 0.7% but was near 8% in PCV-Cas9 transfected cells. Additionally, a 2- to 3-fold increase in the HDR versus indel ratio was observed (Fig. 2f and Supplementary Fig. 4). TIDE analysis^18^ at the top four exonic off-target sites of the *GAPDH* sgRNA revealed no increase in off-targeting effects due to covalent tethering of the ssODN (Supplementary Fig. 5). Of course, off-target effects could be generally reduced in our experiments by making PCV fusions with recently engineered high-fidelity versions of Cas9^19,20^. These data show that our covalent tethering strategy enhances precise gene editing while also increasing absolute HDR efficiencies. In contrast, our attempts to use the recently reported S1mplex donor DNA tethering strategy to insert HiBiT into GAPDH resulted in barely detectable luciferase signal in the HiBiT assay (Supplementary Fig. 6), which is consistent with the authors’ observation that S1mplex resulted in a reduction in HDR from 1% to 0.07% when introducing a 12 bp insertion in HEK-239T cells^11^.

### Tethering enhances HDR in fluorescent reporter cells

**Fig. 3:**
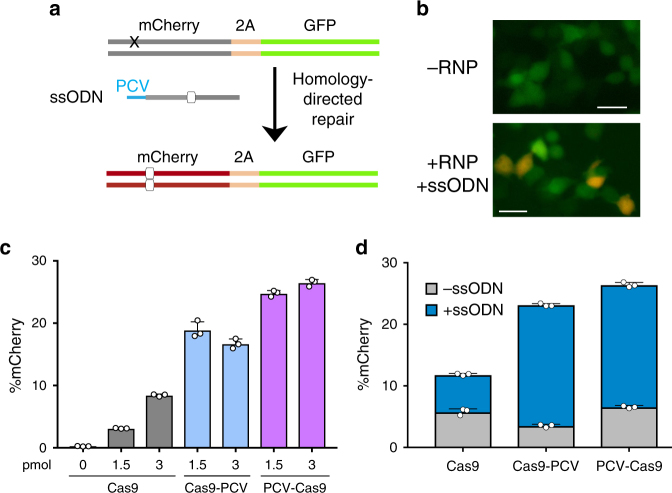
Covalent tethering enhances HDR efficiency in a fluorescent reporter cell line. **a** HEK-293T cells stably expressing a mutant mCherry-GFP reporter are edited by HDR through a frameshift correction, restoring mCherry activity. **b** Representative microscopy images of fluorescent reporter editing. Scale bars are 50 µm. **c** The percent of mCherry positive cells determined by flow cytometry at two different RNP concentrations using a 175 bp ssODN containing the PCV recognition sequence. **d** RNP transfections at 3 pmol in the presence or absence of ssODN. Data are shown as mean+/− SD (*n* = 3). For (**c**) and (**d**), the statistical significance of % mCherry positive cells between PCV-fusions of Cas9 and Cas9 alone was < 0.001, calculated using 2-tailed Student’s *t* test

Next, we aimed to quantify HDR performing a different type of repair. Using an integrated dual fluorescent GFP-mCherry reporter HEK-293T cell line (St. Martin et al.^21^, in the press), we restored mCherry fluorescence by providing an ssODN HDR template to correct a frameshift mutation (Fig. 3a, b). Using flow cytometry, the HDR efficiency measured for cells transfected with Cas9-PCV fusions and PCV+ ssODN was found to be significantly higher than unfused Cas9 at 3 pmol RNP (Fig. 3c). When decreasing the covalent complex concentration, HDR enhancement is 6- to 8-fold, while Cas9-PCV-mediated editing efficiency remains relatively constant. These findings are consistent with the luminescence assay data where lower concentrations result in increased enhancement. We also transfected RNPs without ssODN to account for indel formation that can also produce an in-frame gene. The degree of mCherry restoration without ssODN is relatively constant amongst the Cas9 variants while the contribution when adding ssODN for Cas9-PCV fusions is significantly greater than for Cas9 (Fig. 3d).

## Discussion

We have presented a strategy to improve HDR by covalently tethering the donor DNA to the Cas9 RNP that can easily be incorporated into any Cas9 workflow utilizing RNPs without the need for chemical modification of the ssODN. Moreover, this strategy maintains the native sgRNA to Cas9 interaction and does not require the addition of other proteins, in contrast to recent studies^11^. By simply appending a 13 base pair recognition sequence to the 5′-end of an ssODN and using a Cas9-PCV fusion protein, HDR efficiency can be increased up to 30-fold as measured by three separate approaches. Importantly, significantly lower concentrations of HUH-tagged Cas9 are needed to achieve high efficiencies of HDR, which may be related to the additional positive charges on the PCV moiety enhancing its ability to enter the cell^14^. Collectively, this methodology of covalently tethering the donor DNA template to Cas9 is broadly applicable and effective at enhancing HDR across various cell types and targeting loci, and may be further improved by varying PCV fusion location and optimizing homology arm lengths^22,23^.

Our approach is distinct from other recent ssODN linking methods^11–13^ in that we utilize a ssDNA-binding protein, which forms a covalent link with unmodified DNA. Thus, chemical modification of the ssODN or a unique sgRNA sequence is not required, decreasing the complexity of the reaction and making it scalable for in vivo applications. Our strategy offers significant enhancement of HDR compared to Cas9 alone, without decrements in absolute editing percentages that can occur in other tethering strategies^11,13^. Our methodology results in a 20- to 100-fold increase in HDR frequency relative to the S1mplex method of Carlson-Stevermer et al.^11^ (Supplementary Fig. 6). The lack of a requirement for modified DNA should allow the method to be amenable for use with more complex donor DNA templates such as longer templates or double-stranded templates with single-stranded overhangs. While this method requires the use of Cas9 fusion proteins, the purification steps are identical to those used to purify unfused Cas9. Moreover, the use of additional HUH-tags with different target sequences may allow multiplexed one-pot reactions due to the orthogonality of HUH endonucleases^14^. Furthermore, this technique does not require modulating activity of any endogenous protein as is common with small molecule HDR enhancers. We have, however, shown that different approaches to enhancing HDR can be combined with DNA tethering to further enhance editing efficiency.

## Methods

### Nucleotide sequences

The sgRNA and primer sequences are found in Supplementary Tables 2 and 3. The ssODN sequences can be found in Supplementary Data 1. In ssODNs lacking the PCV recognition sequence, an equal length (13 bp) randomized sequence was added in place so homology arm length was kept consistent. All ssODNs and primers synthesized by Integrated DNA Technologies.

### Construct design

*Streptococcus pyogenes* Cas9 was amplified out of the plasmid pET15_SP-Cas9 (a gift from Niels Geijsen, Addgene plasmid #62731) and inserted in pTD68_SUMO-PCV2 at the BamHI site using Infusion cloning (Clontech) to create C-terminally fused Cas9-PCV. N-terminally fused PCV-Cas9 was constructed using Golden Gate assembly in pTD68_SUMO with insertion of an H4-2 linker between PCV and Cas9^24^. Protein sequences can be found in Supplementary Data 1. Catalytically dead Cas9-PCV (Y96F) was created by QuikChange II site directed mutagenesis kit (Agilent Technologies).

### Protein expression and purification

Bacterial expression of proteins was performed in BL21(DE3) *E. coli* using an autoinduction protocol^25^. Following inoculation, cells were rotated at 300 r.p.m. for 8 h at 37 °C, followed by 24 h at 24 °C. Protein purification was performed using the methods of Anders and Jinek^26^. Cells were pelleted and lysed in 50 mM Tris, 200 mM NaCl, 20% sucrose, pH 7.4. The clarified supernatant was first passed over Ni-NTA resin (Thermo Scientific), washed with 15 column volumes of 50 mM Tris, 200 mM NaCl, 15 mM imidazole, pH 7.4, and eluted with 250 mM imidazole followed by overnight incubation with SUMO protease Ulp1 at 4 °C, while dialyzing into 50 mM HEPES, 150 mM KCl, 10% glycerol, 1 mM DTT, 1 mM EDTA, pH 7.5. Cation exchange chromatography was then performed using a 1 mL HiTrap SP HP column (GE Healthcare), eluting using a gradient from 100 mM to 1 M KCl. Fractions were analyzed by SDS-PAGE and pooled. Protein was concentrated in a 100 kDa MWCO spin concentrator (Amicon). Final protein concentration was measured by Bradford Assay (Bio-Rad) or A_280_ absorbance. Aliquots were stored at −20 °C or flash frozen in dry ice/IPA.

### Covalent DNA attachment to Cas9-PCV

Equimolar amounts of Cas9-PCV and the sequence specific ssODN were incubated at room temperature for 10–15 min in Opti-MEM (Corning) supplemented with 1 mM MgCl_2_. Confirmation of the linkage was analyzed by SDS-PAGE (Supplementary Fig. 7). For the fluorescent oligonucleotide reactions, 1.5 pmol of Alexa 488-conjugated ssDNA (IDT) was incubated with 1.5 pmol Cas9-PCV in the above conditions and separated by SDS-PAGE. Gels were imaged using a 473 nm laser excitation on a Typhoon FLA9500 (GE) at the University of Minnesota Imaging Center (Supplementary Fig. 7).

### In vitro cleavage assay

A pcDNA3-eGFP vector or pcDNA5-GAPDH vector was linearized with BsaI or BspQI (NEB), respectively, and column purified. A concentration of 30 nM sgRNA, 30 nM Cas9, and 1× T4 ligase buffer were incubated for 10 min prior to adding linearized DNA to a final concentration of 3 nM. The reaction was incubated at 37 °C for 1 to 24 h, then separated by agarose gel electrophoresis and imaged using SYBR safe gel stain (Thermo Fisher). The percent cleaved was calculated by comparing densities of the uncleaved band and the top cleaved band using Image Lab software (Bio-Rad).

### Cell culture

HEK-293T, U2-OS, and stable dual-fluorescent HEK-293T cells were cultured in DMEM (Corning) supplemented with 10% FBS (Gibco) and 0.5% penicillin/streptomycin (Gibco). Cells were incubated at 37 °C in 5% CO_2_.

### RNP transfection

Guide RNA sequences were purchased from Integrated DNA Technologies. Guide RNA was formed by mixing equimolar amounts of tracrRNA and crisprRNA in duplex buffer (IDT), heating to 95 °C for 5 min, and cooled on the benchtop. Equimolar amounts of Cas9 and guide RNA were incubated in Opti-MEM supplemented with 1 mM MgCl_2_ for 5–10 min at room temperature. Following RNP formation, equimolar amount of ssODN was added and incubated for 10–15 min at room temperature. Transfections were carried out with RNAiMAX (Invitrogen) using the manufacturer’s suggested protocol in either 96-well plate format with 1.5 pmol RNP/ssODN (10 nM final concentration) and 4 × 10^4^ viable cells per well or 24-well plate using 3 pmol RNP/ssODN (6 nM final concentration) and 2 × 10^5^ viable cells per well unless noted. For SCR7-containing experiments, 1 µM SCR7 (Sigma) was added at the time of transfection. Cells were washed and fresh media was added 24 h later. For S1mplex experiments, the protocol of Carlson-Stevermer et al. was followed, with the exception being the use of RNAiMAX as the transfection reagent^11^.

### Luminescence activity assay

Twenty-four to forty-eight hours post-transfection, luminescence measurements were performed using the Nano-Glo HiBiT lytic assay system (Promega). Briefly, confluent cells were detached and lysed in a buffer containing the recombinant N-terminus of nanoluciferase (LgBiT) and nanoluciferase substrate furimazine in 96-well half-volume plates. Lysates were incubated for 10 min rotating, and luminescence intensities were recorded on a LMax II luminometer (Molecular Devices) at a one second integration time.

### Quantitative PCR

Forty-eight hours post-transfection, cells were spun down at 500 × *g* for 5 min. Genomic DNA was extracted and purified from cell pellets using Purelink genomic DNA purification kit (Invitrogen). In triplicate, SYBR-based qPCR reactions were performed using PowerUp SYBR Green Master Mix (Applied Biosciences) on a Bio-Rad CFX96 real-time thermocycler. Cycle conditions: 95 °C for 2 min; 35 cycles of 95 °C for 15 s and 58 °C for 45 s. Melt curve step at 0.5 °C/min. The data were analyzed on CFX Manager software (Bio-Rad). Relative changes in abundance between HiBiT containing DNA and the GAPDH reference were calculated using the Pfaffl method^17^. Melt curve analysis and primer amplification efficiency measurements on serial diluted DNA templates were also carried out (Supplementary Fig. 3).

### Deep sequencing of target loci

A ~200 bp region encompassing each locus was PCR amplified from isolated genomic DNA with ends containing partial adapters. Amplicons were gel purified and subsequently ligated to full barcoded adaptors. Deep sequencing was performed with an Illumina MiSeq with 2 × 150 bp paired-end reads (Genewiz, Amplicon-EZ). Sequencing reads were analyzed using CRISPResso^27^.

### Off-target editing analysis

Exonic off-target sites for the GAPDH sgRNA were identified using the CRISPR design tool (Zhang Lab, MIT). Regions surrounding the top four sites were amplified from genomic DNA and sequenced (Supplementary Table 4). Tracking of Indel by DEcomposition (TIDE) was employed to identify the indel frequency at each site using a maximum indel size of 33 bp to account for HiBiT insertion^18^.

### S1m-sgRNA assembly and gel shift assays

Creation of the *GAPDH* S1m-sgRNA mirrored the previously published protocol^11^. A DNA template containing the T7 promoter was produced using consecutive rounds of overlap extension PCR with Q5 High-Fidelity polymerase (NEB). The HiScribe T7 RNA Synthesis Kit (NEB) was used to produce the S1m-sgRNA containing the streptavidin aptamer, incubating overnight at 37 °C. The resulting RNA was purified using RNA Clean and Concentrator (Zymo Research) and stored at -80 °C. Gel-shift assays were performed using 20 pmol of S1m-sgRNA, streptavidin, and biotinylated ssODN. Reactions were incubated for 10 min at room temperature and electrophoresed on a 1.5% agarose gel.

### mCherry-GFP HEK-293T editing

Transfections carried out as described above in 24-well plates with 3 pmol of RNP/ssODN unless stated. 48 h post-transfection, cells were detached and centrifuged at 500 × *g* for 5 min. Following aspiration of supernatant, cells were resuspended in 200 µl PBS+1% EDTA and transferred to a 96-well plate. 10,000–100,000 cells were analyzed using flow cytometry on a BD Biosciences FACSCanto II with data collected using BD FACSDiva and compiled using FlowJo (version 10.4). Cells were initially gated based on FSC/SSC and then gated on presence or lack of fluorescent protein expression (Supplementary Fig. 8). In separate experiments, fluorescence microscopy cell images were obtained 24 h post-transfection on an EVOS FL Auto (Life Technologies) using a ×20 objective. GFP and RFP light cubes were used to image GFP and mCherry, respectively. Images were processed using Fiji (version 1.51r).

### Statistical analyses

Data are represented as mean+/− SD (*n* = 3). Experiments were performed multiple independent times with different protein preparations yielding similar trends. Where mentioned in the text and figure legends, statistical tests were performed using two-tailed Student’s *t* test.

### Data availability

Additional supporting data beyond what is in the text and Supplementary Information is available from the authors upon request. Deep sequencing data are available at NCBI SRA accession SRP134004.

## Electronic supplementary material

Supplementary Information

Description of Additional Supplementary Files

Supplementary Data 1
