# Supplementary material for: Increasing Cas9-mediated homology-directed repair efficiency through covalent tethering of DNA repair template
Source: Commun Biol. 2018 May 31;1:54. doi: 10.1038/s42003-018-0054-2 (PMC6123678; doi:10.1038/s42003-018-0054-2)
Supplement: Supplementary file 1 — Supplementary Information [file 42003_2018_54_MOESM1_ESM.pdf]

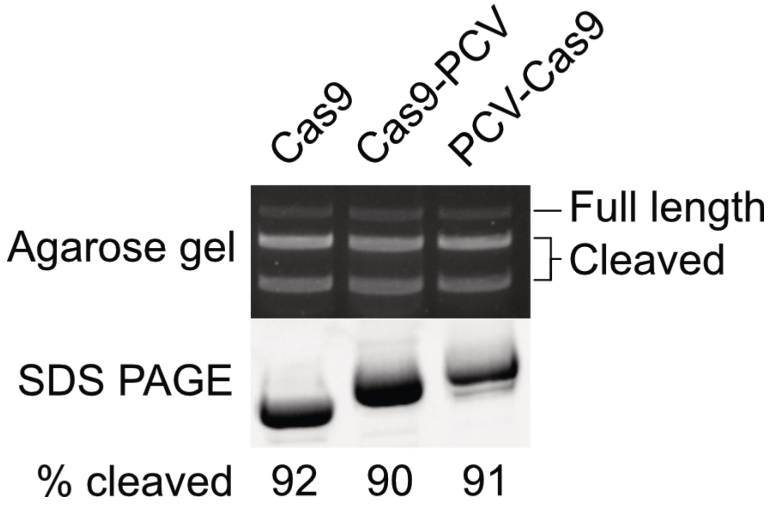

Supplementary Figure 1. Assessing Cas9 fusion *in vitro* cleavage activity. GFP-pcDNA3 DNA was linearized and incubated with RNP targeting GFP for 24 hours and electrophoresed on an agarose gel. The equivalent amount of RNP was also analyzed on SDS-PAGE. The percent of DNA cleaved was calculated using densitometry comparing the top band to the middle band.

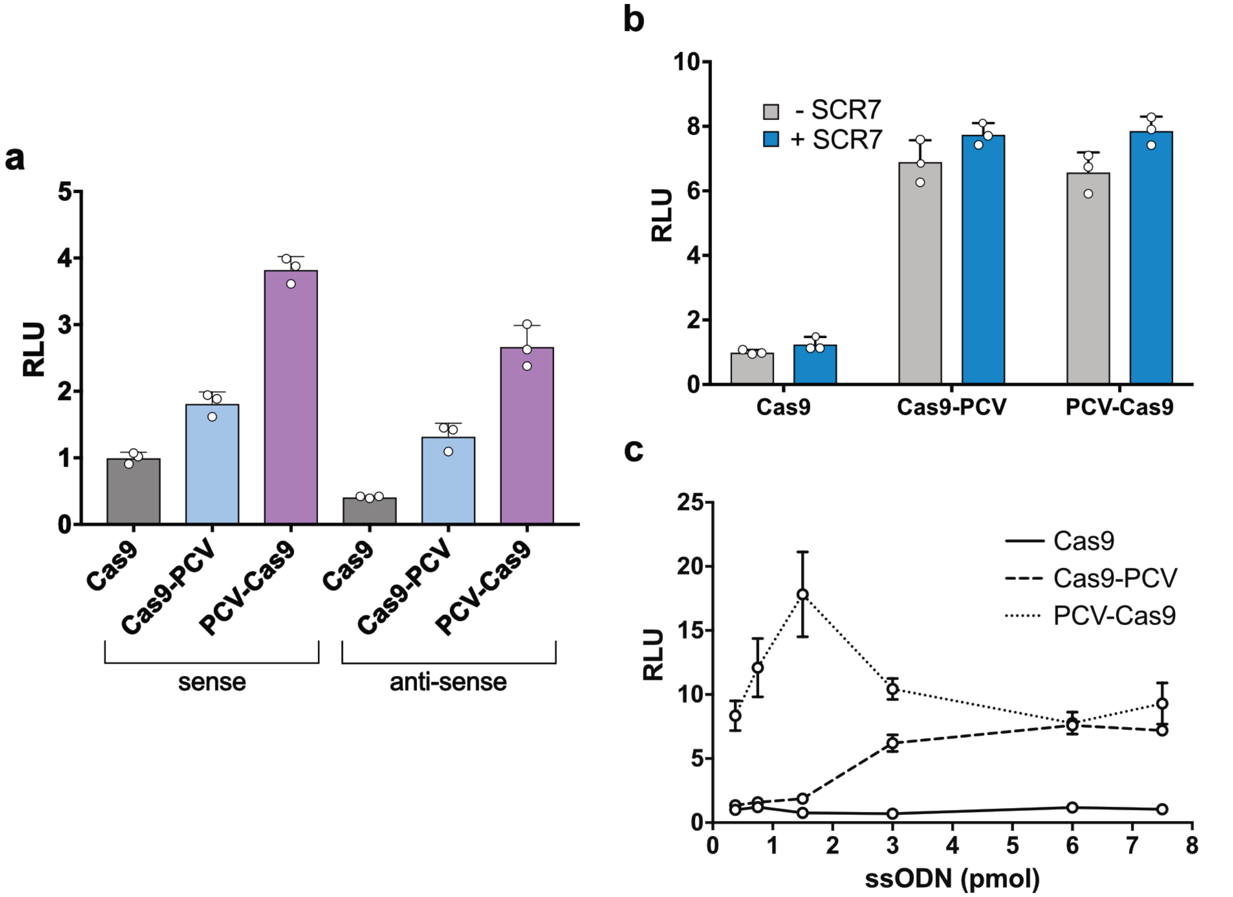

Supplementary Figure 2. Further evidence for robustness of Cas9-PCV fusion. (**a**) The DNA strand sense of the ssODN was reversed (anti-sense) and compared to the standard sense ssODN at 1.5 pmol RNP. (**b**) RNP + ssODN containing PCV recognition sequence were transfected with and without the addition of SCR7. 24 hours post-transfection, cells were washed and replenished with fresh media, and luminescence was assayed another 24 hours later. Readout normalized to Cas9 minus SCR7. Two-tailed Student's t-test P-values: Cas9 and Cas9-PCV ($P > 0.05$), PCV-Cas9 ($P < 0.05$). (**c**) Titration of ssODN while keeping RNP concentration constant at 1.5 pmol. Values in relative luminescence units (RLU). Data are shown as mean +/- SD (n=3).

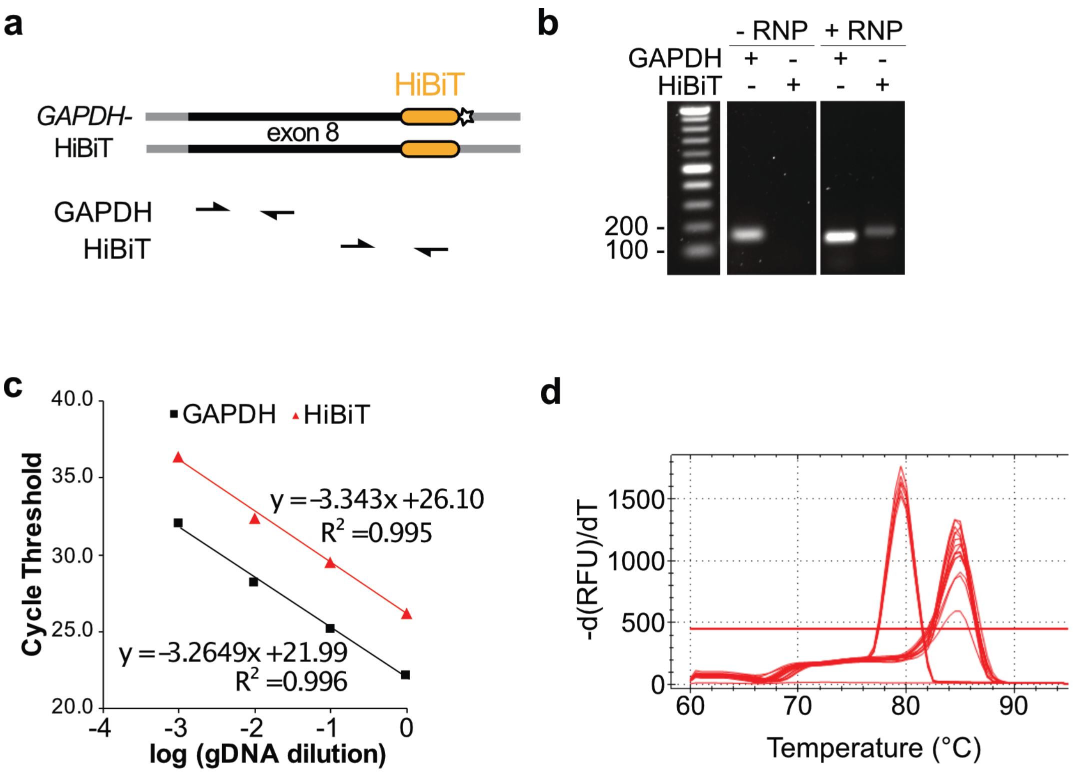

e

DNA source	Target	C_T	Average C_T
Cas9	GAPDH	21.89	
		21.93	21.94±0.06
		22.00	
	HiBiT	25.77	
		25.54	25.32±0.60
		24.64	
Cas9-PCV	GAPDH	22.17	
		22.00	22.07±0.09
		22.05	
	HiBiT	24.52	
		24.52	24.46±0.10
		24.34	
**Change in HDR with Cas9-PCV*:* 1.98 fold***			

* Ratio calculated using Pfaffl Method where *GAPDH* is the reference

Supplementary Figure 3. Quantitative PCR data corroborates luminescence readout. (**a**) Diagram of PCR primer binding sites at the GAPDH locus. (**b**) End-point PCR using both primer pairs on unedited (-RNP) and edited (+RNP) cells. Expected amplicon sizes are 130 bp for GAPDH pair and 187 bp for HiBiT pair. (**c**) Serial dilution

of purified genomic DNA (gDNA) from a pool of edited cells using both primer pairs. Limit of detection is set at 40 cycles. Dynamic range represented by range between first and last values. Best fit line equation shown on graph with corresponding R^2 values. (**d**) Melt curve analysis comparing temperature to change in fluorescence. The first peak corresponds to GAPDH amplicon. (**e**) Calculated cycle threshold values. Relative change in HiBiT amplification is calculated using the Pfaffl method. Primer efficiencies used in calculation determined from (c) were 102% and 99% for GAPDH and HiBiT, respectively.

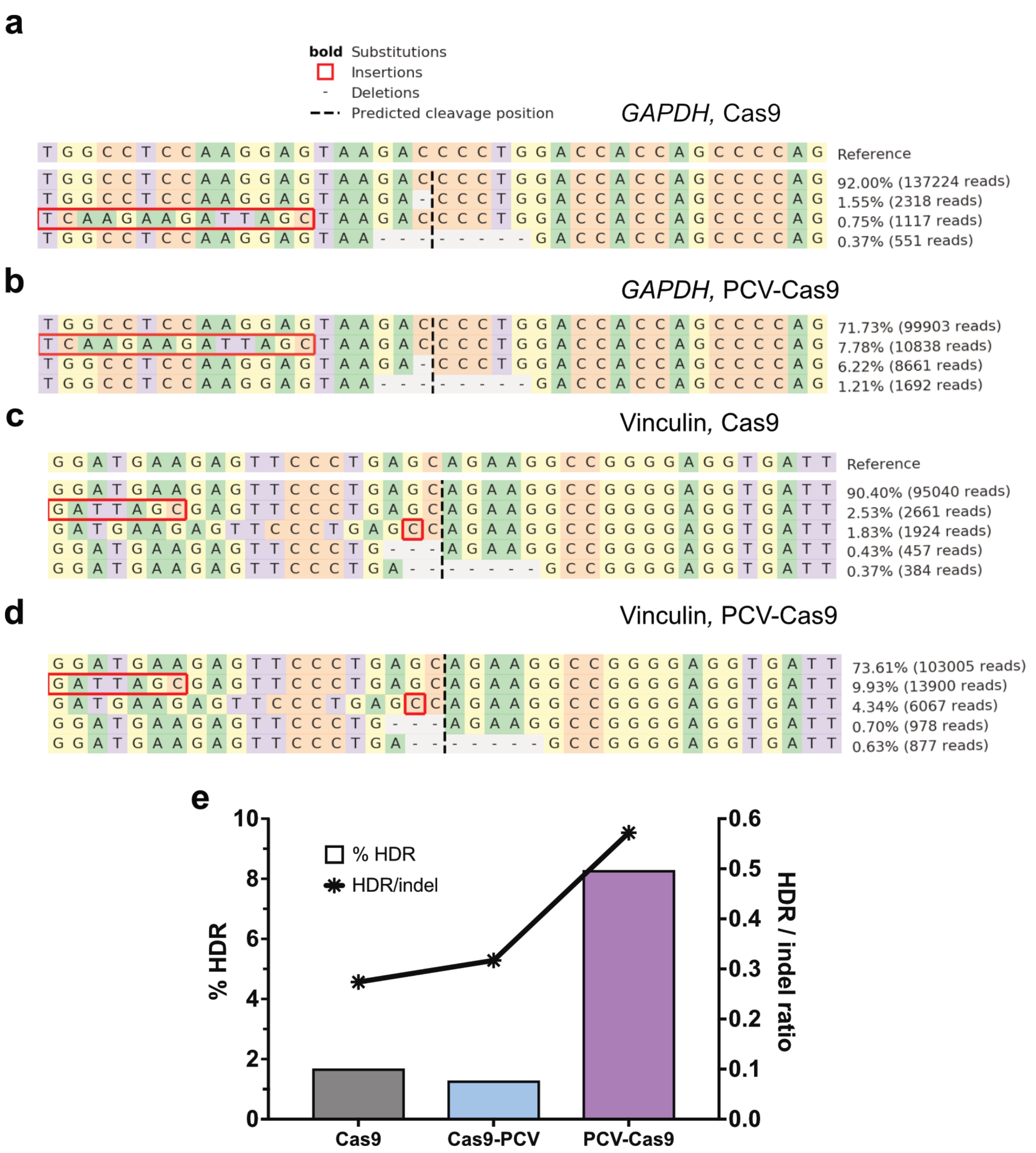

Supplementary Figure 4. Deep sequencing confirmation of HiBiT insertion and HDR enhancement. (**a-d**) Sequencing reads from *GAPDH* and vinculin edited loci. The expected insertion of HiBiT is evident by the red highlighted box. (**e**) HDR frequency and HDR/indel ratio from targeting the vinculin locus.

a

b

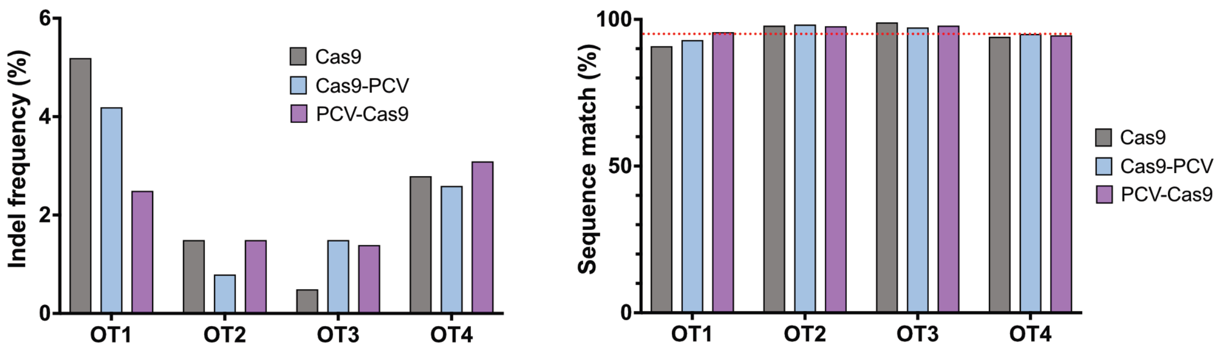

Supplementary Figure 5. No increase in off-target editing with tethered ssODN. The top four predicted exonic off-target sites for the GAPDH sgRNA were analyzed using TIDE. (**a**) The calculated frequency of indel formation for each off-target (OT) site for each Cas9 variant. (**b**) The percentage of perfect sequence matches with the unedited sequence. The 95% limit of detection for TIDE is shown as a red line.

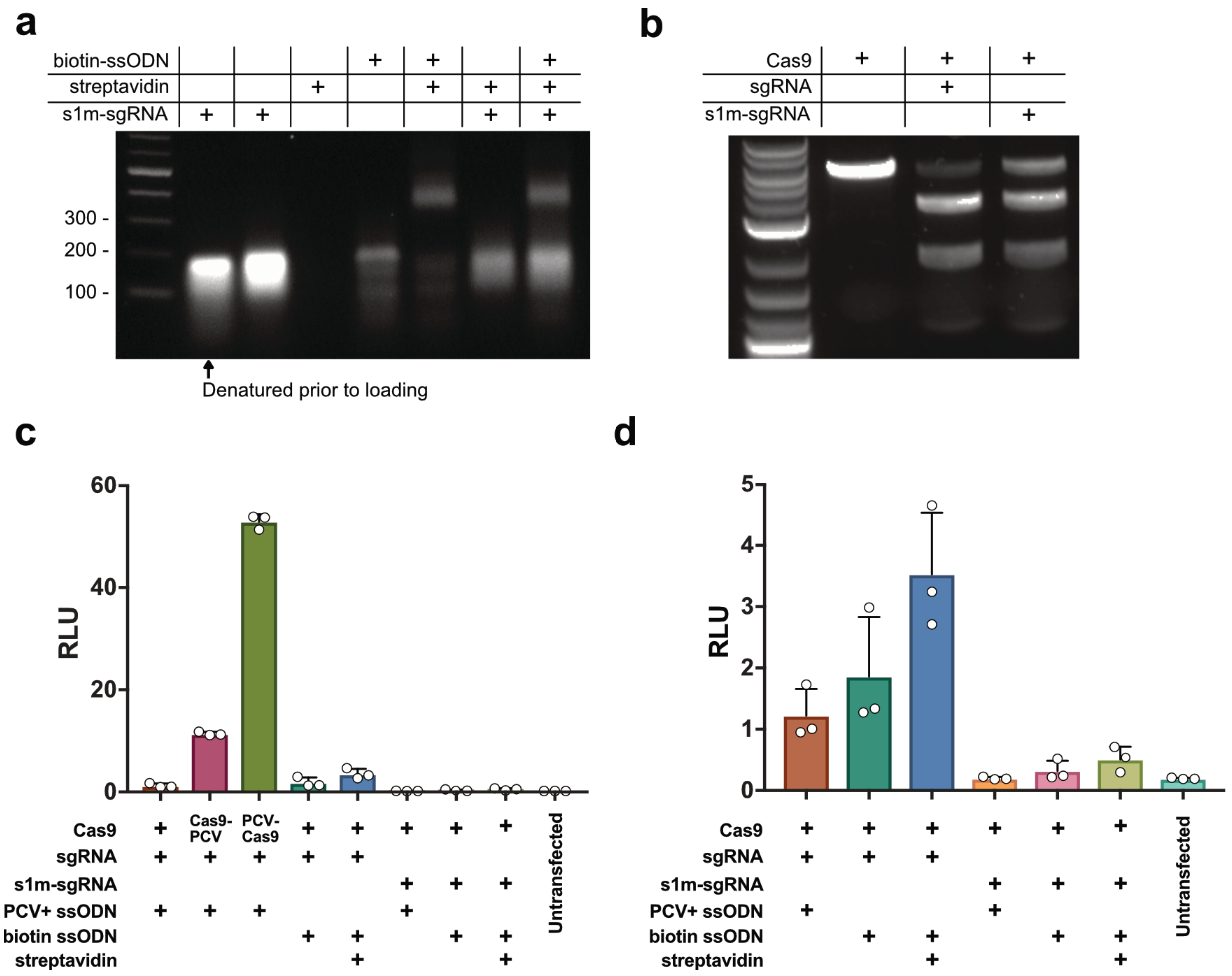

Supplementary Figure 6. Direct comparison to S1mplex tethering strategy. (**a**) Gel shift assay containing differing combinations of components in the S1mplex system. (**b**) An *in vitro* cleavage reaction targeting a linearized vector containing *GAPDH*. Cleavage by Cas9-sgRNA is expected to produce two products. (**c and d**) RNP transfection comparing the Cas9-PCV method to the S1mplex method. (**d**) A more detailed view of the results in (**c**). Values in relative luminescence units (RLU). Data are shown as mean +/- SD (n=3). Experiments were repeated multiple times with similar results.

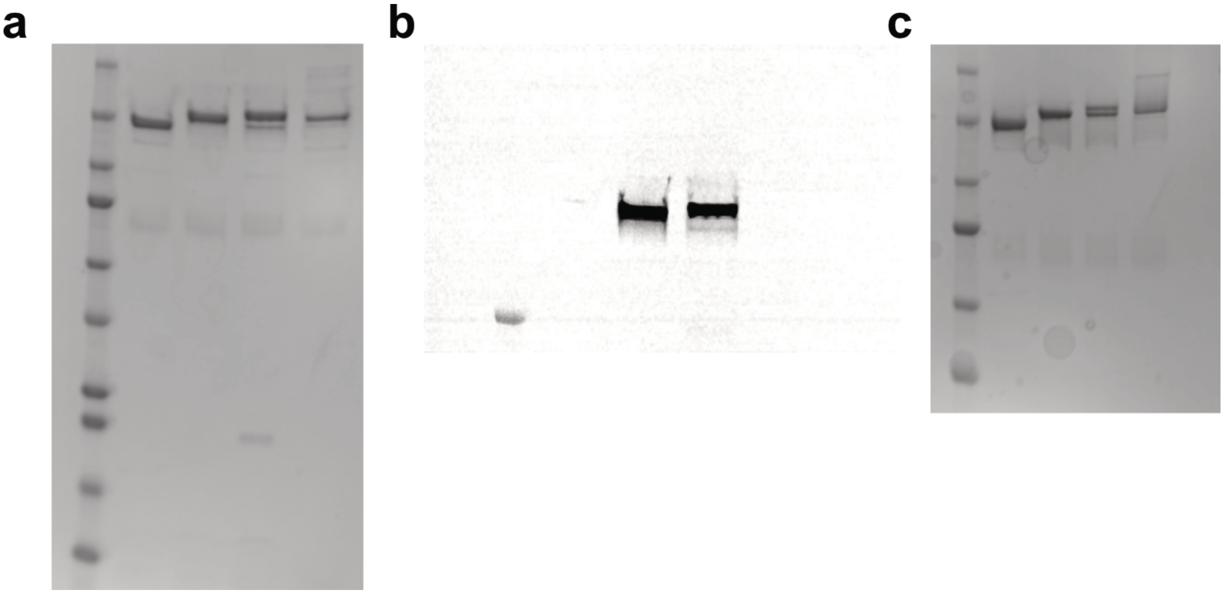

Supplementary Figure 7. Full length gel images. (**a**) Full length gel from Figure 1b, upper panel. (**b**) Original gel scan obtained from fluorescent gel scanner from Figure 1b, lower panel. (**c**) Full length gel image from Figure 1c.

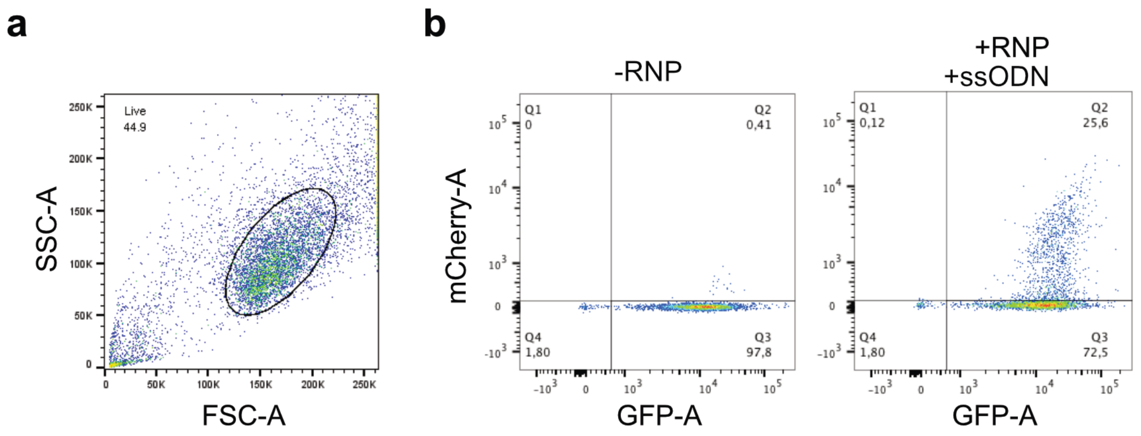

Supplementary Figure 8. Flow cytometry gating methodology. (**a**) Flow cytometry plot of gating used to select live cells based on the forward scatter (FSC) and side scatter (SSC). 10,000 events are measured. (**b**) Flow cytometry plots of gated live cells comparing GFP intensity to mCherry intensity in transfected (+RNP +ssODN) versus untransfected cells (-RNP). Gating is based on untransfected cells.

Supplementary Table 1. Deep sequencing results

		HDR %	Indel %	HDR/Indel	HDR fold change
GAPDH	Cas9	0.7	5.2	0.135	1
	Cas9-PCV	4.0	14.6	0.274	5.71
	PCV-Cas9	7.9	18.1	0.436	11.29
Vinculin	Cas9	1.7	6.2	0.274	1
	Cas9-PCV	1.3	4.1	0.317	0.76
	PCV-Cas9	8.3	14.5	0.572	4.88

Supplementary Table 2. Sequences of primers

Primer	DNA sequence (5'-to-3')
GAPDH_F	CTCCCACCTTTCTCATCCAAG
GAPDH_R	ACATCACCCCTCTACCTCC
HiBiT_F	GAGACTGGCTCTTAAAAAGTGC
HiBiT_R	GCTAATCTTCTTGAACAGCCG
Cas9-PCV-Y96F_F	CGATCAGCAGAACAAAGAATTTTGTAGCAAAGAAGGCAAC
Cas9-PCV-Y96F_R	GTTGCCTTCTTTGCTACAAAATTCTTTGTTCTGCTGATCG
HTS_GAPDH_F (includes adaptor)	ACACTCTTTCCCTACACGACGCTCTTCCGATCT ctgacaactcttttcatcttct
HTS_GAPDH_R (includes adaptor)	ACACTCTTTCCCTACACGACGCTCTTCCGATCT aaagtgcagggtctggcg
HTS_Vinculin_F (includes adaptor)	ACACTCTTTCCCTACACGACGCTCTTCCGATCT atgagcttgctcctcccaaacc
HTS_Vinculin_R (includes adaptor)	GACTGGAGTTCAGACGTGTGCTCTTCCGATCT tcactacttaccttgctggacc
OT1_F	CTCCTTGGAATCGTCGTCCT
OT1_R	CGGCGCTGCAGCTTTGAAAA
OT2_F	AGCATGAGATTCGGACTGTAG
OT2_R	CCACAAAGCTTCGGTTGACT
OT3_F	GGCAGTTTCTAGGACTGTCC
OT3_R	GGGTGCCTTTCCTAGTACCA
OT4_F	GAGAGCTGATGATGCTCTGG
OT4_R	TGGTCCATGAATGACGGAAGA

Supplementary Table 3. Guide RNA sequences

sgRNA target	Sequence (5'-to-3')	PAM
GAPDH	CCTCCAAGGAGTAAGACCCC	AGG
Vinculin	TGAAGAGTTCCCTGAGCAGA	AGG
mCherry	TGGCCCCTCACCCTTCGCCT	GGG
GFP	CTCGTGACCACCCTGACCTA	CGG

Supplementary Table 4. GAPDH off-target sites

Site	Off-target sequence	PAM	Locus	Transcript
**OT1**	CCTCCAACTAGTAAGAACCC	GGG	chr11:-71951342	NM_005169
**OT2**	CATCCAGGGACCAAGACCCC	CAG	chr7:-148981327	NM_001195220
**OT3**	TCTCCAAGGAGTCAGTCCCC	CAG	chr2:+47302091	NM_020458
**OT4**	CCTGCAGGGAGGGAGACCCC	AGG	chr12:-133578192	NM_001256279

Mismatches from sgRNA sequence are labeled in red
