# Supplementary material for: Increasing Cas9-mediated homology-directed repair efficiency through covalent tethering of DNA repair template
Source: Commun Biol. 2018 May 31;1:54. doi: 10.1038/s42003-018-0054-2 (PMC6123678; doi:10.1038/s42003-018-0054-2)
Supplement: Supplementary file 2 — Description of Additional Supplementary Files [file 42003_2018_54_MOESM2_ESM.docx]

**Description of Additional Supplementary Files**

File Name: Supplementary Data 1

Description: Sequences of ssODNs and proteins
